# Nucleus Accumbens Core Dopamine D2 Receptor-Expressing Neurons Control Reversal Learning but Not Set-Shifting in Behavioral Flexibility in Male Mice

**DOI:** 10.3389/fnins.2022.885380

**Published:** 2022-06-28

**Authors:** Tom Macpherson, Ji Yoon Kim, Takatoshi Hikida

**Affiliations:** ^1^Laboratory for Advanced Brain Functions, Institute for Protein Research, Osaka University, Suita, Japan; ^2^Medical Innovation Center, Graduate School of Medicine, Kyoto University, Kyoto, Japan

**Keywords:** nucleus accumbens, behavioral flexibility, medium spiny neuron, reversal learning, set-shifting, decision-making, striatum, response inhibition

## Abstract

The ability to use environmental cues to flexibly guide responses is crucial for adaptive behavior and is thought to be controlled within a series of cortico-basal ganglia-thalamo-cortical loops. Previous evidence has indicated that different prefrontal cortical regions control dissociable aspects of behavioral flexibility, with the medial prefrontal cortex (mPFC) necessary for the ability to shift attention to a novel strategy (set-shifting) and the orbitofrontal cortex (OFC) necessary for shifting attention between learned stimulus-outcome associations (reversal learning). The nucleus accumbens (NAc) is a major downstream target of both the mPFC and the OFC; however, its role in controlling reversal learning and set-shifting abilities is still unclear. Here we investigated the contribution of the two major NAc neuronal populations, medium spiny neurons expressing either dopamine D1 or D2 receptors (D1-/D2-MSNs), in guiding reversal learning and set-shifting in an attentional set-shifting task (ASST). Persistent inhibition of neurotransmitter release from NAc D2-MSNs, but not D1-MSNs, resulted in an impaired ability for reversal learning, but not set-shifting in male mice. These findings suggest that NAc D2-MSNs play a critical role in suppressing responding toward specific learned cues that are now associated with unfavorable outcomes (i.e., in reversal stages), but not in the suppression of more general learned strategies (i.e., in set-shifting). This study provides further evidence for the anatomical separation of reversal learning and set-shifting abilities within cortico-basal ganglia-thalamo-cortical loops.

## Introduction

Behavioral flexibility refers to the adaptation of behavior in response to changes in the internal or external environment, and is a critical skill for survival in our everchanging world (Brown and Tait, 2014; Uddin, 2021). Indeed, impaired behavioral flexibility (also known as behavioral rigidity) is a major characteristic of several neurodegenerative disorders, including Alzheimer’s, Huntington’s, and Parkinson’s diseases, as well as psychiatric conditions, including schizophrenia, autism spectrum disorders, and obsessive–compulsive disorders (Cools et al., 2001, 2022; Hong and Rebec, 2012; Chen et al., 2013; Dajani and Uddin, 2015; Gruner and Pittenger, 2017; Macpherson and Hikida, 2019). Depending on the situation, flexible behavior is thought to require different types of learning, although in experimental psychology these have generally been grouped into paradigms investigating the ability to switch attention between learned stimulus–response–outcome (S–R–O) contingencies (reversal learning) or the ability to shift attention from a learned strategy to a new strategy (set-shifting) (Izquierdo and Jentsch, 2012; Brown and Tait, 2014; Izquierdo et al., 2017). To study the neural substrates underlying such types of learning, researchers have developed several behavioral tasks that typically require rodents, non-human primates, or humans to dynamically alter their behavioral responses to environmental cues signaling changing outcomes (Izquierdo and Belcher, 2012; Izquierdo and Jentsch, 2012; Izquierdo et al., 2019). One such task that has gained popularity in rodent studies has been the attentional set-shifting task (ASST). The advantage of this task is its ability to measure discriminative goal-directed learning, as well as both reversal learning and set-shifting forms of behavioral flexibility, within the same paradigm (Brown and Tait, 2014; Tait et al., 2014; Heisler et al., 2015). However, a limitation is that the ASST often uses only two possible choices, making it difficult to assess whether response errors during reversal stages are the result of perseveration or rather a more general cognitive impairment.

Flexible goal-directed behavior is thought to be collaboratively controlled by cognitive/associative and limbic information processing cortico-basal ganglia-thalamo-cortical loop circuits (Balleine, 2019; Macpherson et al., 2021). At the origin of these circuits, cortical structures have been revealed to play dissociative roles in controlling behavioral flexibility, with inactivation of the orbitofrontal cortex (OFC) reported to disrupt reversal learning, but not set-shifting (Bohn et al., 2003; Bissonette et al., 2008; Floresco et al., 2008; Ghods-Sharifi et al., 2008; Torregrossa et al., 2008; Graybeal et al., 2011; Morris et al., 2016; Izquierdo, 2017; Groman et al., 2019), and inactivation of the medial prefrontal cortex (mPFC) resulted in the opposite phenotype (Birrell and Brown, 2000; Bissonette et al., 2008; Morris et al., 2016). While it is important to note that the precise definitions of these cortical regions remain controversial, based on the injection sites used in these studies it appears that spatially separate regions of the cortex control distinct aspects of behavioral flexibility.

Downstream of projections from both the OFC and mPFC, the nucleus accumbens (NAc) of the ventral striatum has also been implicated in behavioral flexibility (Floresco et al., 2006, 2009; Haluk and Floresco, 2009; Cui et al., 2018; Li et al., 2018; Ma et al., 2020). Within this region, neurons can largely be divided into two subpopulations: dopamine D1 or D2 receptor-expressing medium spiny neurons (D1-/D2-MSNs). While both NAc Core D1- and D2-MSNs receive an approximately equivalent amount of inputs from the OFC and the mPFC, both cell types receive especially dense innervation from the mediolateral OFC and prelimbic mPFC (Li et al., 2018; Ma et al., 2020). Previous research has indicated that while NAc D1-MSNs are implicated in Pavlovian reward-related learning (Flagel et al., 2007; Hikida et al., 2010; Lobo et al., 2010; Calipari et al., 2016; Macpherson and Hikida, 2018; Soares-Cunha et al., 2019), NAc D2-MSNs appear to contribute to motivation, aversion, and reversal learning (Macpherson et al., 2014, 2016; Hikida et al., 2016; Soares-Cunha et al., 2016, 2018, 2022). Additionally, it has recently been revealed that altered neurotransmission in NAc D1- and D2-MSNs is able to bidirectionally control gene expression within the mPFC (Hikida et al., 2020), indicating that the NAc may itself be able to modulate mPFC-related cognitive functions such as the ability for attentional set-shifting. However, despite this, the exact role of NAc D1- and D2-MSNs in controlling attentional set-shifting is still unclear.

Here, we chronically blocked the neurotransmitter release specifically from either NAc Core D1- or D2-MSNs and investigated the effect on discrimination learning, reversal learning, and set-shifting abilities within an ASST for mice. Our findings indicate that while NAc Core D2-MSNs contribute to reversal learning, they are not implicated in the control of set-shifting, providing additional evidence that these two types of behavioral flexibility are controlled by separate neurocircuits. Additionally, we reveal that impairment of reversal learning following NAc Core D2-MSN neurotransmitter release inhibition is associated with a reduced decision latency in the error trials, suggesting that these neurons may contribute to the inhibition of learned S–R–O associations that have become unfavorable, but not in the general inhibition of undesirable decision-making strategies.

## Materials and Methods

### Animals

Male NAc D1-/D2-MSN neurotransmission-blocked mice (D1-/D2-MSN-Blocked) and wildtype (WT) controls, aged between 10 and 16 weeks, were generated using the TRE-TeNT-GFP transgenic mice on a C57BL/6 background, as previously described (Hikida et al., 2010; Macpherson et al., 2016). Tetanus toxin (TeNT) is a bacterial toxin that blocks the release of neurotransmitters from the presynaptic terminal of the neurons in which it is expressed by cleaving the vesicle-associated membrane protein VAMP2 (Schiavo et al., 1992; Wada et al., 2007). In TRE-TeNT-GFP mice, the expression of TeNT and green fluorescent protein (GFP) is under the control of tetracycline responsive element (TRE) and is driven by the interaction of TRE with tetracycline transactivator (tTA) (Yamamoto et al., 2003; Wada et al., 2007).

In both WT and TRE-TeNT-GFP mice, tTA was specifically expressed in either NAc D1-MSNs or D2-MSNs, which is known to coexpress the peptides substance P (SP) or Enkephalin (ENK), respectively (Gerfen et al., 1990; Lu et al., 1997), by bilateral microinjections of a recombinant adeno-associated virus (AAV) construct (AAV2-SP-tTA or AAV2-ENK-tTA) into the NAc (AP: +1.5 mm, ML: ±0.8 mm, DV +3.5 mm; 500 nl infused at 50 nl/min; spread of ±0.5 mm in each area) under anesthesia (90 mg/kg Ketamine and 20 mg/kg Xylazine, i.p. injection). This resulted in persistent blocking of neurotransmitter release from either NAc D1- or D2-MSNs of TeNT mice (D1-MSN-Blocked: *n* = 8, D2-MSN-Blocked: *n* = 9), but had no effect on WT (*n* = 8, per group) mice, an effect that has been previously been electrophysiologically validated (Hikida et al., 2010). Post-surgery, mice were provided with an anti-inflammatory drug (10 mg/kg Rimadyl, Zoetis, Florham Park, NJ, United States) in their drinking water for 1 week and left in their home cages for 3–4 weeks for adequate viral expression and surgical recovery.

**FIGURE 1:**
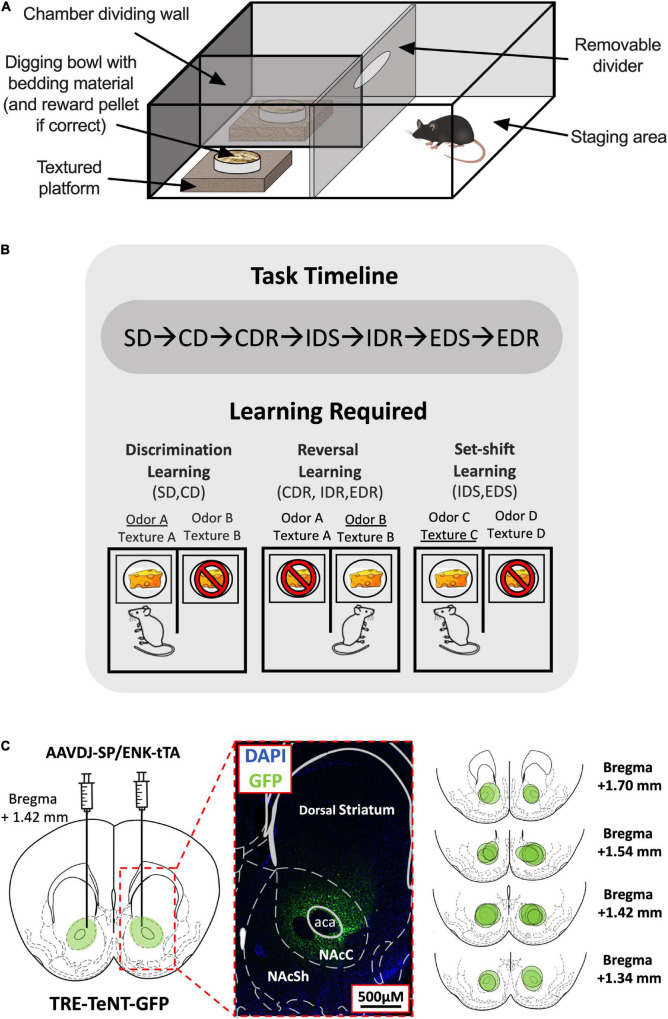
Attentional set-shifting task (ASST) experimental setup. **(A)** Layout of the ASST apparatus. **(B)** Timeline of the ASST (top). The three types of learning tested in the ASST and the stages in which they are required (bottom). Examples of correct (indicated by cheese) and incorrect responses (indicated by cheese with a stop sign) and their associated reward-signaling (underlined) and non-reward-signaling (not underlined) cues during each stage type are shown. **(C)** Virus injection site (left) and magnified histological example of TeNT-GFP expression within the NAc Core region indicated by dotted red lines (middle). The virus spread area for each D1-/D2-MSN-Blocked mouse is indicated by separate green circles in the NAc (right). SD, simple discrimination; CD, compound discrimination; CDR, compound discrimination reversal; IDS, intradimensional shift; IDR, intradimensional shift reversal; EDS, extradimensional shift; EDR, extradimensional shift reversal.

Mice were housed in groups of 2–4 and were maintained on a 12-h light/dark schedule (lights on at 8 a.m.) at a temperature of 24 ± 2°C and humidity of 50 ± 5% controlled room. Beginning 3 days before the commencement of experiments, mice were food restricted to 85% of their free-feeding weight on standard lab chow, with water available *ad libitum*. Behavioral experiments were performed between the hours of 10 a.m. to 6 p.m. Following the completion of experiments, virus infusion locations were histologically verified by immunohistochemical investigation of GFP expression (Figure 1C), and two mice were excluded due to misaligned injection sites. All animal handling procedures and use of viruses were approved by the animal research committees of the Kyoto University Graduate School of Medicine (approval ID: MedKyo17071) and the Institute for Protein Research, Osaka University (approval ID: 29-02-1).

### Apparatus

The ASST was performed in a sound-attenuating experimental room using a square opaque acrylic box [45 cm (L) × 45 cm (W) × 30 cm (D)] divided halfway down the front center line by an opaque barrier to create two equal-sized chambers and a rectangular staging area that had a removable acrylic divider placed horizontally 15 cm from the back wall (Figure 1A). In each of the two chamber areas, a shallow square polyethylene platform [15 cm (L) × 15 cm (W) × 5 cm (D)] was added, and on top of each platform was placed a circular plastic weighing dish (7 cm diameter) acting as a digging bowl. The platforms were either left as they were or wrapped in one of the five materials (styrofoam, corrugated cardboard, metal wire, sandpaper, and bubble wrap) to provide six different tactile cues. The digging bowls were filled with woodchips bedding that had been infused with one of the six different odors (coffee, cinnamon, rosemary, garlic, ground ginger, and nutmeg). Odorless sucrose pellets (Dustless Precision Pellets,^®^ Sucrose, Unflavored, 20 mg, Bio-Serv, Flemington, NJ, United States) were used for rewards, and it was verified before the experiment commencement that mice were unable to detect the location of a baited bowl at a greater chance level (50 ± 10% accuracy across a total of 50 trials using two woodchips filled bowls; one baited and one not) when no location cues (odor/texture) were provided.

### Behavioral Testing

#### Shaping

Day 1: The location of the reward was trained by placing mice in the testing apparatus containing two digging bowls (one on each side) with five sucrose pellets placed on top of the unscented woodchip bedding. Once mice had consumed all the pellets and the bowls were rebaited until the mice had collected 40 pellets.

Day 2: Mice were trained to dig to collect the reward by hiding a sucrose pellet under the unscented woodchip bedding in each of the digging bowls. Once the rewards had been consumed, the bowls were rebaited until 40 pellets had been collected.

Day 3: Mice were trained to dig in scented digging bowls placed atop textured platforms, with each odor and platform type presented an equal number of times in a pseudo-random order until 40 pellets had been collected.

#### Attentional Set-Shifting Task Paradigm

The ASST paradigm was based on a previously established protocol (Young et al., 2010) with minor adjustments. At the start of each trial, mice were placed into the staging area at the rear of the testing chamber and the plastic divider was removed to allow access to the two digging bowls, one of which contained a pellet reward. In the first four trials, mice were allowed access to the baited bowl irrespective of whether a response error occurred, allowing them to learn the cue-outcome contingency (in such cases, an error was still recorded). In subsequent trials, mice were blocked access to the chamber containing the baited bowl using the divider following a response error and were immediately returned to the staging area until the start of the next trial. In correct trials, mice were similarly returned to the staging area immediately following consumption of the reward. Trials were continued until the mouse had made six consecutive correct choices, or until a cutoff of 40 trials occurred, at which point they progressed to the next stage of the task. Digging was defined as the mouse’s front paws or nose entering the bedding medium. If digging did not occur within 5 min of the trial start, the trial was designated as an omission and did not contribute toward the trials to criterion or incorrect latency measures.

The ASST is composed of seven different stages: simple discrimination (SD), compound discrimination (CD), compound discrimination reversal (CDR), intradimensional shift (IDS), intradimensional shift reversal (IDR), extradimensional shift (EDS), and extradimensional shift reversal (EDR) (Table 1). Each stage consisted of repeated trials that assessed the ability of mice to use a specific cue dimension (odor or texture) to discriminate between rewarded and non-rewarded digging bowls. In the SD stage, mice were exposed to only one dimension that could be used for discrimination, whereas, during the CD stage, both dimensions were present but the relevant dimension (used for discrimination) was unchanged from the previous (SD) stage. In the IDS stage, the relevant dimension remained the same as in the previous stages (SD, CD, and CDR), but new odor and platform cues were introduced, requiring the mouse to relearn the cue-outcome contingencies, albeit using the same strategy. In the EDS stage, the relevant dimension was changed and new odor and platform cues were introduced. Finally, in the reversal stages (CDR, IDR, and EDR), the correct and incorrect cues were reversed for the relevant dimension. While the order of the stages was never changed, relevant dimensions and cue orders were randomized across animals. For each trial, the latency to dig was recorded by an experimenter with a stopwatch, beginning when the divider was lifted and ending when the mouse began digging.

**TABLE 1:** Examples of correct and incorrect odor and platform material cues used in each stage of the attentional set-shifting task (ASST).

Task	Dimension		Cue		Example cues	
	Relevant	Irrelevant	Correct	Incorrect	Correct	Incorrect
Simple discrimination (SD)	Odor		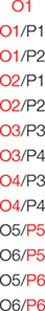	O2	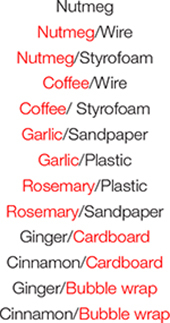	Coffee
Compound discrimination (CD)	Odor	Platform		O2/P1		Coffee/Wire
				O2/P2		Coffee/Styrofoam
CD reversal (CDR)	Odor	Platform		O1/P1		Nutmeg/Wire
				O1/P2		Nutmeg/Styrofoam
Intradimensional set-shift (IDS)	Odor	Platform		O4/P3		Rosemary/Sandpaper
				O4/P4		Rosemary/Plastic
IDS reversal (IDR)	Odor	Platform		O3/P3		Garlic/Sandpaper
				O3/P4		Garlic/Plastic
Extradimensional set-shift (EDS)	Platform	Odor		O5/P6		Ginger/Bubble Wrap
				O6/P6		Cinnamon/Bubble Wrap
EDS reversal (EDR)	Platform	Odor		O5/P5		Ginger/Cardboard
				O6/P5		Cinnamon/Cardboard

*Correct cues at each stage are indicated in red. Potential odor cues included: nutmeg, coffee, garlic, rosemary, ginger, and cinnamon. Potential platform material cues included: wire, styrofoam, plastic, sandpaper, cardboard, and bubble wrap.*

Testing was performed over 2–3 days, with stages presented in the following order in daily blocks: Day 1: SD, CD, and CDR; Day 2: IDS, IDR, EDS, and EDR; or Day 1: SD, CD, and CDR; Day 2: IDS and IDR; Day 3: EDS and EDR (Figure 1B).

### Statistical Analyses

Trials to criterion and total errors were collected for each stage; however, as these two measures are correlated and analysis of either produced the same results, only trials to criterion are reported [as previously described (Birrell and Brown, 2000; Young et al., 2010)]. Response latencies (seconds) were separated into the mean latencies to perform a correct or an incorrect response (mean correct/incorrect latency). The total amount of omissions per session was also recorded.

The data were found to be normally distributed (Shapiro–Wilk tests; *p* > 0.05) and the assumption of homogeneity of variance was not violated (Levene’s tests; *p* > 0.05). Data were analyzed separately for D1- and D2-MSN-Blocked groups (including their respective WTs) initially using repeated measures of three-way ANOVAs with *stage* (SD, CD, CDR, IDS, IDR, EDS, and EDR) as a within-subjects variable and *group* (D1-/D2-MSN-Blocked or WT) and *dimension change* (odor-to-platform and platform-to-odor) as between-subject variables. The influence of dimension change was also checked separately for D1- and D2-MSN-Blocked groups using univariate three-way ANOVAs with the EDS stage as the dependent variable and *group* (D1-/D2-MSN-Blocked or WT) and *dimension change* (odor-to-platform and platform-to-odor) as independent variables. As no significant main effect or interaction of *dimension change* was found in all the analyses (see Supplementary Table 1), dimensions were grouped together for all the subsequent analyses as well as in the presented graphs. D1- and D2-MSN-Blocked groups were then reanalyzed separately using repeated measures of two-way ANOVAs with *stage* (SD, CD, CDR, IDS, IDR, EDS, and EDR) as the within-subjects variable and *group* (D1-/D2-MSN-Blocked or WT) as the between-subject variable. Additionally, to validate the formation of attentional sets, trials to criterion in IDS vs. EDS stages were analyzed separately in D1- and D2-MSN-Blocked groups using repeated measures of two-way ANOVAs with *stage* (IDS and EDS) as the within-subjects variable, and *group* (D1-/D2-MSN-Blocked or WT) as the between-subject variable. *Post-hoc* analyses of significant effects were performed using the Bonferroni test. Correlations between trials to criterion and mean incorrect latency were analyzed using the Pearson’s correlation coefficients. Statistical significance was considered to be *p* < 0.05. All statistical analyses are presented in Supplementary Table 1.

### Immunohistochemistry

Following the completion of experiments, mice were anesthetized (90 mg/kg Ketamine and 20 mg/kg Xylazine, i.p. injection) and then transcardially perfused with cold 4% paraformaldehyde in 0.1 M phosphate buffer (pH 7.4) (Nacalai Tesque, Kyoto, Japan). Brains were removed from the skull and soaked in 30% sucrose in phosphate-buffered saline (PBS; pH 7.4) for 3 days until completely submerged, frozen at −20°C with compound medium (Tissue-Tek O.C.T. compound, Sakura Finetech, Tokyo, Japan), and then sliced into 30 μm coronal sections using a cryostat (Leica CM1860, Leica Biosystems, Wetzlar, Germany). Free-floating sections in PBS were subjected to immunohistochemistry [as previously described (Ohishi et al., 1994)] using a rabbit polyclonal anti-GFP primary antibody (A-11122, ThermoFisher Scientific, Waltham, MA, United States) diluted (1:500) in PBS and a fluorescent secondary antibody conjugated to Alexa 488 (Life Technologies, Newark, CA, United States) also diluted (1:200) in PBS. Sections were mounted with Vectashield containing DAPI (Vector Laboratories, CA, United States) and images were captured using a Keyence BZ-X810 fluorescence microscope (Keyence, Osaka, Japan).

## Results

### Histology

Immunohistochemical staining of GFP found expression of the viral vector to be largely restricted to the NAc Core, with minimal spillover to NAc Shell or dorsal striatal regions (Figure 1C).

### Task Validation

**FIGURE 2:**
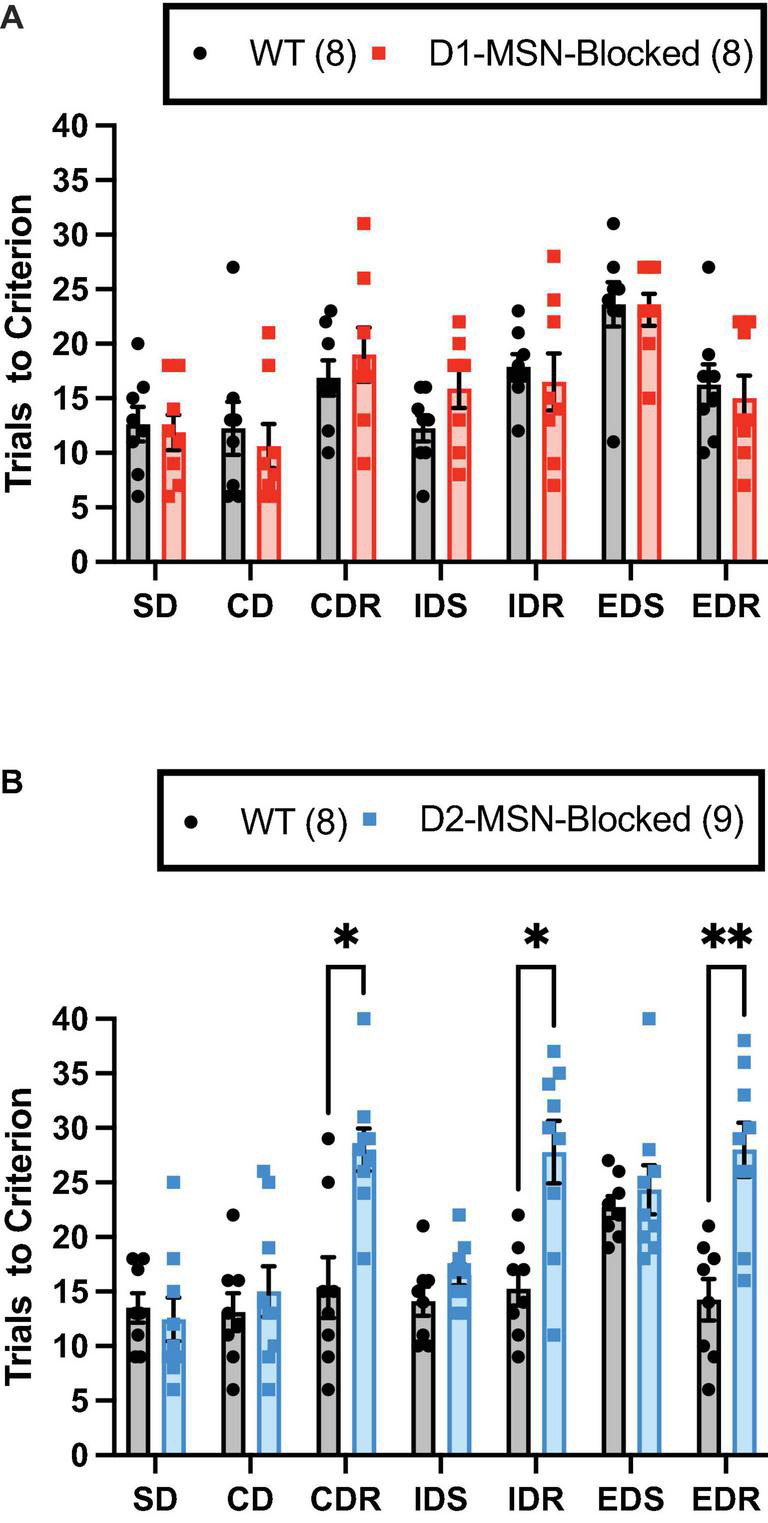
Reversal learning, but not set-shift or discrimination learning, requires neurotransmission in NAc Core D2-MSNs. NAc Core D1-MSN-Blocked (*n* = 8) **(A)** and D2-MSN-Blocked (*n* = 9) **(B)** mice did not differ from wildtype (WT) mice (*n* = 8, respectively) in their ability to perform discrimination (SD and CD) and set-shifting (IDS and EDS) stages of the attentional set-shifting task (ASST). However, D2-MSN-Blocked, but not D1-MSN-Blocked, mice were impaired in their ability for reversal learning during all three reversal stages (CDR, IDR, and EDR). Bars represent mean ± SEM; Bonferroni *post-hoc* tests (*p < 0.05, **p < 0.01).

The ability of WT and D1-/D2-MSN-Blocked mice to perform the ASST was measured. A significant main effect of the stage on trials to criterion was observed in both D1-MSN-Blocked [Figure 2A; *F*_(6,84)_ = 10.78, *p* < 0.001] and D2-MSN-Blocked [Figure 2B; *F*_(6,90)_ = 5.34, *p* < 0.001] groups, indicating that mice’s performance varied across the different stages. Additionally, a comparison of performance on the IDS vs. the EDS stage for internal validation of attentional set formation (Young et al., 2010) revealed a significant main effect of the stage on trials to criterion for both D1-MSN-Blocked [Supplementary Figure 1A; *F*_(1,14)_ = 24.06, *p* < 0.001] and D2-MSN-Blocked [Supplementary Figure 1B; *F*_(1,15)_ = 27.85, *p* < 0.001] groups. Poorer performance on the ED stage by both groups indicated that all animals were able to successfully form an attentional set to the internal stimulus dimension. Analysis of omissions found no significant main effect of stage or genotype, and no stage × genotype interaction for both D1- and D2-MSN-Blocked groups, indicating that inhibition of neurotransmitter release from NAc Core D1- or D2-MSNs likely did not alter task engagement.

### Attentional Set-Shifting Task Performance

The D2-MSN-Blocked group, but not the D1-MSN-Blocked group, demonstrated a significant main effect of genotype [Figure 2B; *F*_(1,15)_ = 37.20, *p* < 0.001], as well as a significant stage × genotype interaction [Figure 2B; *F*_(6,90)_ = 5.34, *p* < 0.001], on trials to criterion. Subsequent *post-hoc* analyses using Bonferroni’s multiple comparison test revealed that the D2-MSN-Blocked mice took a significantly greater amount of trials to reach criterion on all reversal stages (CDR, IDR, and EDR), but not discrimination (SD and CD) or set-shift stages (IDS and EDS), than WT controls (Figure 2B). These findings suggest that blockade of neurotransmitter release from NAc Core D2-MSNs was able to impair the ability for reversal learning.

Investigation of the mean correct latency revealed a significant main effect of the stage in both D1-MSN-Blocked [Supplementary Figure 2A; *F*_(6,84)_ = 4.34, *p* < 0.01] and D2-MSN-Blocked groups [Supplementary Figure 2B, *F*_(6,90)_ = 3.96, *p* < 0.01]. However, no significant main effect of genotype or interaction between stage and genotype were found (Supplementary Table 1). Thus, in all mice, correct response times appeared to vary across different stages.

**FIGURE 3:**
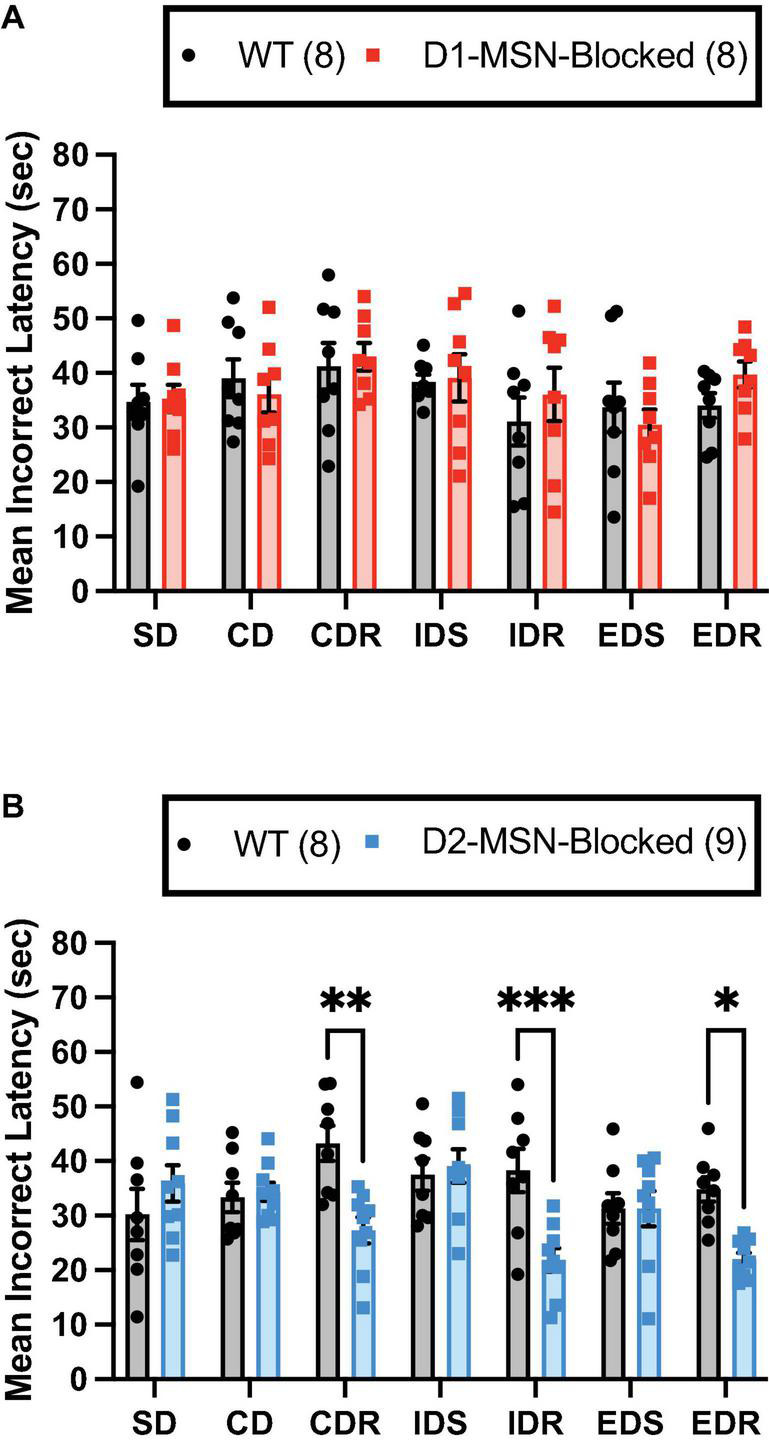
NAc Core D2-MSN neurotransmitter release blockade reduces the latency to make response errors during reversal learning. NAc Core D1-MSN-Blocked (*n* = 8) **(A)** and D2-MSN-Blocked (*n* = 9) **(B)** mice did not differ from wildtype (WT) controls (*n* = 8) in their mean latency to make response errors in discrimination (SD and CD) and set-shifting (IDS and EDS) stages of the attentional set-shifting task (ASST). However, neurotransmission release blockade in NAc Core D2-MSNs, but not D1-MSNs, resulted in a shorter latency to make response errors during reversal learning stages (CDR, IDR, and EDR) compared with WT controls. Bars represent mean ± SEM; Bonferroni *post-hoc* tests (*p < 0.05, **p < 0.01, ***p < 0.001).

Finally, for the mean incorrect latency, a significant main effect of the stage was found for both D1-MSN-Blocked [Figure 3A; *F*_(6,84)_ = 2.51, *p* < 0.05] and D2-MSN-Blocked [Figure 3B; *F*_(6,90)_ = 2.35, *p* < 0.05] groups, suggesting that, with the mean correct latency, incorrect response times varied across different stages. Additionally, in the D2-MSN-Blocked, but not D1-MSN-Blocked, group a significant main effect of genotype [Figure 3B; *F*_(1,15)_ = 11.43, *p* < 0.001], as well as a significant stage × genotype interaction [Figure 3B; *F*_(6,90)_ = 4.67, *p* < 0.001] was found. *Post-hoc* Bonferroni’s multiple comparison tests revealed that incorrect response times were shorter in all reversal stages (CDR, IDR, and EDR), but not in discrimination (SD and CD) or set-shift stages (IDS and EDS), than in WT controls (Figure 3B). Subsequent Pearson’s correlation coefficient analysis of reversal stages (CDR, IDR, and EDR) demonstrated that the incorrect latency and, for the most part, the mean correct latency were significantly negatively correlated with trials to criterion in D2-MSN-Blocked and WT mice (Supplementary Figures 4A–F). These findings suggest that as response time slows down, accuracy in reversal stages of the ASST increases. Moreover, it is plausible that the reduced response time in error trials in reversal stages may underlie the impaired performance in these stages, with less time for cognitive deliberation resulting in a reduction in response accuracy.

## Discussion

Previous studies have demonstrated that dissociable aspects of behavioral flexibility are controlled by separate subregions of the frontal cortex, with the OFC and mPFC revealed to be integral for reversal learning and set-shifting abilities, respectively (Birrell and Brown, 2000; Bohn et al., 2003; Bissonette et al., 2008; Floresco et al., 2008; Ghods-Sharifi et al., 2008; Graybeal et al., 2011; Spellman et al., 2021). These findings raise the possibility that reversal learning and set-shifting abilities may be controlled by separate information processing pathways within the cortical-basal ganglia-thalamo-cortical loop. Alternatively, given that both the OFC and mPFC send major projections to D1- and D2-MSNs of the NAc (Li et al., 2018; Ma et al., 2020), it is plausible that the NAc could play an important role in controlling both types of behavioral flexibility. Here, using an odor and texture cue-based ASST in mice, we revealed that while NAc D2-MSNs contribute to reversal learning ability by inhibiting incorrect responding, they are not implicated in the control of set-shifting.

### NAc D1- and D2-MSNs Are Not Involved in Discrimination Learning Ability in the Attentional Set-Shifting Task

Our study found that neurotransmission blocking in NAc Core D1- and D2-MSNs did not alter the ability for discrimination learning in the initial acquisition stage of the ASST. While these findings are consistent with a previous study from our group indicating that signaling in NAc D1- and D2-MSN is not necessary for the acquisition of a spatial discrimination task (Macpherson et al., 2016), they contrast with other studies reporting NAc D1-MSN activity to be necessary for spatial and visual discrimination tasks (Hikida et al., 2010; Nishioka et al., 2021). These findings may be explained by differences in the complexity of the tasks used. It has been suggested that NAc signaling becomes necessary when task requirements are ambiguous or require considerable cognitive or physical effort (Floresco, 2015; Macpherson et al., 2021). Indeed, in visual discrimination tasks disrupted by NAc inactivation, animals were required to inhibit responses to known cues and respond only at random cues (Nishioka et al., 2021), or to respond correctly at one of the five possible response windows (five-choice serial reaction time test) (Christakou, 2004; Pezze et al., 2007). Similarly, in NAc inactivation-impaired spatial learning tasks, animals were required to navigate through up to eight possible locations in a radial arm maze (Floresco et al., 1997; Gal et al., 1997). Despite the distracting influence of task-irrelevant cues in the current ASST study, the discrimination learning stages are considerably simpler than the above-described visual and spatial discrimination studies, requiring mice to choose between only two possible options. As such, they match with the previous evidence demonstrating NAc inactivation to have no effect on the performance of discrimination tasks with only two-to-four locations (Castañé et al., 2010; Macpherson et al., 2016) or visual cues (Floresco et al., 2006).

### Evidence for Nucleus Accumbens Control of Behavioral Flexibility

The role of the NAc in reversal learning is complicated, with NAc inactivation studies often reporting apparently conflicting results depending on differences in the method of NAc manipulation used, the region of the NAc targeted, the species tested, and the type of reversal learning measured. In rats, NMDA receptor (NMDAR) blockade of either the NAc Core or Shell with the NMDAR antagonist AP5, an effect likely to inhibit the neural activity of these regions, was reported to impair reversal learning in a spatial operant task (Ding et al., 2014), while performance in a similar task was found to be unaltered following quinolinic acid lesions of either the NAc Core or Shell (Castañé et al., 2010). In a spatial T-maze task, dopamine depletion of the NAc using 6-hydroxydopamine lesions has been reported to disrupt reversal learning (Taghzouti et al., 1985); however, reversal learning in an operant probabilistic task and a deterministic task was found to be disrupted by pharmacological inactivation of the NAc Shell, but not Core, using the GABA receptor agonists baclofen and muscimol (Dalton et al., 2014). In non-human primates, ibotenic acid lesions of the NAc were reported to impair visual reversal learning, but, in contrast to inactivation studies in rodents, did not alter spatial reversal learning (Stern and Passingham, 1995). Finally, in humans, fMRI analysis of subjects performing visual reversal learning tasks has similarly identified NAc activation during reversal error responses (Cools et al., 2002).

In set-shifting studies, baclofen and muscimol inactivation of the NAc Core of rats has been revealed to disrupt switching from spatial to visual cue-based strategies in a radial arm-based task, with NAc Shell inactivation oppositely facilitating set-shifting (Floresco et al., 2006). Similarly, infusion of NMDAR antagonist AP5 into the NAc Core, but not Shell, impaired set-shifting from visual to spatial cue-based strategies in an operant task (Ding et al., 2014).

Despite their dissimilar findings, the above-described studies, as well as this and previous studies from our group (Yawata et al., 2012; Macpherson et al., 2016), support an important role for NAc neurons in mediating behavioral flexibility.

### NAc D2-MSNs Mediate Reversal Learning Ability in the Attentional Set-Shifting Task

In the ASST, neurotransmission blocking in NAc D2-MSNs, but not D1-MSNs, was demonstrated to impair performance in reversal learning stages, where two learned S–R–O associations were switched. This finding supports those of previous studies from our group and others indicating signaling in NAc D2-MSNs to be critical for reversal learning in both visual and spatial discrimination tasks (Yawata et al., 2012; Macpherson et al., 2016; Cui et al., 2018). In general, these findings are also supported by previous studies investigating the effect of pharmacological manipulation of dopamine receptors on reversal learning. Intra-NAc Core infusion of a D2R agonist, but not a D1R agonist or a D1R or D2R antagonist, was revealed to disrupt reversal learning in a visual discrimination task in rats (Haluk and Floresco, 2009). Whereas, a more recent study reported that intra-NAc Core infusion of a D2R antagonist, but not a D1R antagonist, was able to improve reversal learning in a visual discrimination task by reducing perseverative errors (Sala-Bayo et al., 2020). Given that D2Rs are Gi protein-coupled receptors that act to inhibit the D2-MSNs in which they are expressed (Shen et al., 2008), these findings suggest that the activity of NAc Core D2-MSNs contributes to the ability for reversal learning. Interestingly, constitutive deletion of D2Rs has also been shown to reduce reversal learning ability in ASST (DeSteno and Schmauss, 2009), odor discrimination (Kruzich and Grandy, 2004), and visual discrimination (Morita et al., 2016) tasks. Thus, it is possible that disturbance of normal NAc D2-MSN signaling, either by increased or reduced activity, may be sufficient to disrupt reversal learning ability. However, the possible influence of D2R deletion in areas outside of the NAc on reversal learning in these tasks cannot be discounted.

Finally, a potential limitation of the current study is that it included only a single rather than serial reversal stages. Previous studies have revealed that disruption of the OFC is able to impair the initial, but not serial, reversal stages in serial reversal odor discrimination tasks (Schoenbaum et al., 2002, 2003), suggesting that reversal learning deficits may not persist following repeated training. While it is not clear how neurotransmitter release inhibition in NAc Core D2-MSNs may affect performance on serial reversal learning stages in an ASST, previous work from our group has revealed that, in NAc Core D2-MSN-Blocked mice, impaired reversal learning in an initial reversal stage of a serial reversal place discrimination task was gradually restored to the level of controls across repeated reversals (Macpherson et al., 2016). In this study, we speculated that other brain regions, potentially the dorsal striatum, may be able to compensate for impaired reversal learning ability following repeated training across serial reversal stages.

### Reversal Learning Impairment in NAc Core D2-MSN NeurotransmIssion Blocked Mice Is Associated With Faster Incorrect Responding Toward Outdated Reward Cues

Reversal learning deficits following neurotransmission blocking in NAc Core D2-MSNs were found to be associated with a reduced average latency to make an incorrect response, but no change in the average latency to make a correct response when compared with WT controls. These data suggest that signaling from NAc D2-MSNs plays an important role in increasing the decision-making time concerning whether to respond to previously correct and now outdated learned S–R–O associations, potentially helping to reduce the likelihood of incorrect responding. In support of the importance of NAc Core D2-MSNs in response inhibition, a recent study revealed that intra-NAc Core infusion of the D2R antagonist raclopride, but not the D1 antagonist SCH-23390, selectively reduced early perseverative errors in a visual cue-based serial reversal-learning task (Sala-Bayo et al., 2020). Oppositely, intra-NAc Core infusion of the D2 agonist quinpirole has been found to increase perseverative error responses in five-choice serial reaction time tasks (Pezze et al., 2007). These findings suggest that signaling from NAc Core D2-MSNs may be able to bidirectional control perseveration. In the current study, it was not possible to directly assess perseverative errors due to the choice of only two response options; however, in a previous study by our group, neurotransmitter release inhibition from NAc Core D2-MSNs resulted in an increase in perseverative, but not general, errors in serial reversal learning stages of a four-choice spatial discrimination task (Macpherson et al., 2016).

Inhibition of perseverative responding in reversal learning tasks is suggested to require information feedback concerning response errors (Klanker et al., 2013). Both animal and computational data have revealed that D2Rs play a critical role in such signaling of response errors during visual and probabilistic reversal learning tasks, allowing learning from losses (Alsiö et al., 2019). These findings are also congruent with recent evidence from our group demonstrating NAc D2-MSNs to be critical for the future avoidance of non-rewarded cues following response errors (Nishioka et al., 2021). Studies in humans also appear to support the role of the NAc in response inhibition, with fMRI analysis identifying robust activation of the NAc during the final reversal error of a visual reversal learning task, immediately before a switch in responding toward the correct visual cue (Cools et al., 2002).

Overall, our data add to a growing literature demonstrating the importance of NAc D2-MSNs in inhibiting incorrect behavioral responses, likely by providing necessary feedback or response errors.

### NAc Core MSNs Are Not Involved in the Set-Shifting Ability in the Attentional Set-Shifting Task

A major finding of our study was that the neurotransmission blocking of NAc Core D1- and D2-MSNs did not alter the ability for set-shifting in the ASST. These findings contrast with the previous studies that demonstrate bilateral inactivation of the NAc Core, as well as disconnection of prefrontal and thalamic inputs to the NAc Core, to impair the set-shifting ability (Floresco et al., 2006; Block et al., 2007). Similarly, the same group revealed that intra-NAc administration of pharmacological agents acting at D1R and D2R modulate the set-shifting ability. Haluk and Floresco (2009) reported that a D1R, but not D2R, antagonism as well as D2R, but not D1R, agonism disrupted the set-shifting ability from a visual to a spatial cue-based strategy. However, another group investigating the role of D2Rs in behavioral flexibility found no effect of constitutive D2R deletion on set-shifting from odor to texture cue-based strategies in the ASST (DeSteno and Schmauss, 2009).

It is unclear why impairments in the set-shifting ability reported in the above-described studies of NAc inactivation were not observed in the current study. However, it should be noted that our study differs from these studies in several methodological factors. First, while previous studies of the NAc and set-shifting have tended to use rats, our study used mice. Second, in contrast to previous studies that used visual or spatial cues to guide responding, in our task, mice were required to utilize odor and tactile cues. Thus, it is possible that while activity in NAc Core D2-MSNs is necessary for switching to or from visual or spatial cue-based strategies, these neurons may not be necessary for strategy switching based on odor and tactile cues. As described in the previous section (see Section Evidence for Nucleus Accumbens Control of Behavioral Flexibility), similar outcome differences in studies utilizing different species or cue modalities are not uncommon, and further investigation is likely necessary to identify how behavioral flexibility based on information from various modalities may be differentially controlled within the NAc of various species. Finally, in previous studies, NAc subregions and cell types were inactivated acutely by intracranial infusions of dopamine or GABA receptor ligands, or anesthetics. In contrast, our study utilized a chronic NAc Core D1-/D2-MSN inactivation method. It is possible that such chronic inactivation may result in neuroplastic compensatory mechanisms, such as a different brain region being recruited, that allow the animal to regain the ability for set-shifting. However, if this is the case, it is unclear why reversal learning remained impaired. Future studies utilizing transient-cell-type-specific inactivation methods, such as Cre-dependent inhibitory opsins or artificial receptors in transgenic D1-/D2-Cre lines, may help to elucidate this question.

Studies on humans have indicated that the dorsal striatum and its inputs from the mPFC may contribute significantly to the control of set-shifting. The fMRI analysis of healthy controls found significant activation of dorsal frontal-striatal regions during set-shifting stages of a visual discrimination task, whereas OCD patients demonstrating the dysfunctional set-shifting ability showed no such activation (Gu et al., 2008). Conversely, carriers of a DRD2/ANNK1-Taqla polymorphism that results in reduced D2 receptors, particularly in the dorsal striatum, demonstrated impaired set-shifting performance in a visual cue-guided reward learning task and decreased functional connectivity between mPFC and dorsal striatal regions (Noble et al., 1997; Stelzel et al., 2010). In positron emission tomography (PET) studies, increased dopamine release has been observed in the dorsal striatum during set-shifting (Monchi et al., 2006), while reduced dopamine in the dorsal striatum during the early stages of Parkinson’s disease is associated with the impaired set-shifting ability (Lewis et al., 2005; Cools, 2006; Kehagia et al., 2010). Interestingly, while treatment of early-stage Parkinson’s disease patients with levodopa reverses set-shifting dysfunction, it has been found to impair performance reversal learning tasks, potentially by excessive stimulation of DA receptors in the ventral striatum which is generally less prone to dopamine depletion during the early stage of the disease (Swainson et al., 2000; Cools, 2006; Kehagia et al., 2010). These studies, alongside our finding that NAc Core D2-MSN neurotransmission blocking impairs reversal learning but not set-shifting, suggest a functional dissociation in the striatal regions controlling different aspects of behavioral flexibility, with an OFC-NAc D2-MSN pathway potentially mediating reversal learning and an mPFC-dorsal striatum potentially mediating the set-shifting ability. Future studies using D1- and D2-MSN-specific neurotransmission blocking in various subregions of the dorsal striatum will likely help to identify the precise striatal circuits responsible for set-shifting.

## Data Availability Statement

The raw data supporting the conclusions of this article will be made available upon reasonable request.

## Ethics Statement

The animal study was reviewed and approved by Animal research committees of Kyoto University Graduate School of Medicine and the Institute for Protein Research, Osaka University.

## Author Contributions

TM and JK carried out the experiments, analyzed the data, and wrote the manuscript. TM and TH conceived the original idea and supervised the research. All authors read and approved the final manuscript.

## Conflict of Interest

The authors declare that the research was conducted in the absence of any commercial or financial relationships that could be construed as a potential conflict of interest.

## Publisher’s Note

All claims expressed in this article are solely those of the authors and do not necessarily represent those of their affiliated organizations, or those of the publisher, the editors and the reviewers. Any product that may be evaluated in this article, or claim that may be made by its manufacturer, is not guaranteed or endorsed by the publisher.

## Supplementary Material

Supplementary Figure 1 Mice demonstrate successful formation of attentional sets. D1-MSN-Blocked (*n* = 8), D2-MSN-Blocked (*n* = 9), and WTs (*n* = 8, per group) took significantly more trials to reach the criterion in the extradimensional shift (EDS) stage than the intradimensional shift (IDS) stage, indicating all animals were able to successfully form an attentional set to the internal stimulus dimension that resulted in poorer performance when the set was shifted. Bars represent mean ± SEM; Bonferroni *post-hoc* tests (**p* < 0.05, ***p* < 0.01).

Supplementary Figure 2 Mean latencies to make correct responses in the attentional set-shifting task (ASST) were unaffected by neurotransmission release inhibition from NAc Core MSNs. NAc Core D1-MSN-Blocked (*n* = 8) **(A)** and D2-MSN-Blocked (*n* = 9) **(B)** mice did not significantly differ from wildtype (WT) controls (*n* = 8) in their mean latency to make a correct response during discrimination [simple discrimination (SD) and compound discrimination (CD)], reversal [compound discrimination reversal (CDR), intradimensional set-shift reversal (IDR), and extradimensional set-shift reversal (EDR)], and set-shifting (IDS and EDS) stages of the ASST. Bars represent mean ± SEM.

Supplementary Figure 3 Omission trials in the attentional set-shifting task (ASST) following NAc Core D1- and D2-MSN neurotransmitter release inhibition. The total amount of omission trials in discrimination (SD and CD), reversal (CDR, IDR, and EDR), and set-shifting (IDS and EDS) stages of the ASST did not significantly differ between WT (*n* = 8 per group) mice and NAc Core D1-MSN-Blocked (*n* = 8) **(A)** or D2-MSN-Blocked (*n* = 9) **(B)** mice. Bars represent mean ± SEM.

Supplementary Figure 4 Mean incorrect latency was negatively correlated with trials to criterion in reversal stages. In both WT (*n* = 8) **(A,C,E)** and NAc D2-MSN-Blocked (*n* = 9) **(B,D,F)** mice, the mean incorrect latency was negatively correlated with trials to criterion in all reversal stages (CDR, IDR, and EDR). Additionally, the mean correct latency was negatively correlated with trials to criterion in IDR and EDR stages in WT mice **(C,E)** and CDR and EDR stages in NAc Core D2-MSN-Blocked mice **(B,F)**. Lines of best fit have been fitted to each correlation plot using simple linear regressions. Additionally, Pearson’s *r* values and the statistical significance of each correlation are presented.
