# Supplementary material for: Nucleus Accumbens Core Dopamine D2 Receptor-Expressing Neurons Control Reversal Learning but Not Set-Shifting in Behavioral Flexibility in Male Mice
Source: Front Neurosci. 2022 Jun 28;16:885380. doi: 10.3389/fnins.2022.885380 (PMC9275008; doi:10.3389/fnins.2022.885380)
Supplement: Supplementary file 1 [file Table_1.docx]

**Supplementary Table S1. Statistical analyses in the ASST**

| **ANOVA** | **Data** | **Subject** | **Factors** | **F(df)** | **Sig** |
| --- | --- | --- | --- | --- | --- |
| Repeated Measures 3-Factor ANOVA | Trials To Criterion | D1-MSN-Blocked & WT | Genotype | F_(1,12)_= 0.31 | ns (0.59) |
|  |  |  | Dimension | F_(1,12)_= 1.44 | ns (0.25) |
|  |  |  | Genotype * Dimension | F_(1,12)_= 3.01 | ns (0.11) |
|  |  |  | **Stage** | **F_(6,72)_= 11.09** | ***p* < 0.001** |
|  |  |  | Stage * Genotype | F_(6,72)_= 0.74 | ns (0.62) |
|  |  |  | Stage * Dimension | F_(6,72)_= 0.92 | ns (0.49) |
|  |  |  | Stage * Genotype * Dimension | F_(6,72)_= 0.49 | ns (0.81) |
| Repeated Measures  3-way ANOVA | Trials To Criterion | D2-MSN-Blocked & WT | **Genotype** | **F_(1,13)_= 36.43** | ***p* < 0.001** |
|  |  |  | Dimension | F_(1,13)_= 0.01 | ns (0.92) |
|  |  |  | Genotype * Dimension | F_(1,13)_= 1.40 | ns (0.26) |
|  |  |  | **Stage** | **F_(6,78)_= 8.66** | ***p* < 0.001** |
|  |  |  | **Stage * Genotype** | **F_(6,78)_= 5.01** | ***p* < 0.001** |
|  |  |  | Stage * Dimension | F_(6,78)_= 0.53 | ns (0.79) |
|  |  |  | Stage * Genotype * Dimension | F_(6,78)_= 0.87 | ns (0.52) |
| Univariate  3-way ANOVA | Trials To Criterion  (EDS only) | D1-MSN-Blocked & WT | Dimension | F_(1,12)_= 0.00 | ns (0.96) |
|  |  |  | Genotype * Dimension | F_(1,12)_= 2.10 | ns (0.17) |
| Univariate  3-way ANOVA | Trials To Criterion  (EDS only) | D2-MSN-Blocked & WT | Dimension | F_(1,12)_= 0.03 | ns (0.87) |
|  |  |  | Genotype * Dimension | F_(1,12)_= 1.41 | ns (0.26) |
| Repeated Measures  3-way ANOVA | Mean Correct Latency | D1-MSN-Blocked & WT | Genotype | F_(1,12)_= 0.02 | ns (0.90) |
|  |  |  | Dimension | F_(1,12)_= 2.87 | ns (0.12) |
|  |  |  | Genotype * Dimension | F_(1,12)_= 2.67 | ns (0.13) |
|  |  |  | **Stage** | **F_(6,72)_= 5.33** | ***p* < 0.001** |
|  |  |  | Stage * Genotype | F_(6,72)_= 1.23 | ns (0.30) |
|  |  |  | Stage * Dimension | F_(6,72)_= 0.94 | ns (0.47) |
|  |  |  | Stage * Genotype * Dimension | F_(6,72)_= 1.91 | ns (0.09) |
| Repeated Measures  3-way ANOVA | Mean Correct Latency | D2-MSN-Blocked & WT | Genotype | F_(1,13)_= 0.22 | ns (0.65) |
|  |  |  | Dimension | F_(1,13)_= 0.20 | ns (0.67) |
|  |  |  | Genotype * Dimension | F_(1,13)_= 0.69 | ns (0.42) |
|  |  |  | **Stage** | **F_(6,78)_= 3.88** | ***p* < 0.01** |
|  |  |  | Stage * Genotype | F_(6,78)_= 0.84 | ns (0.54) |
|  |  |  | Stage * Dimension | F_(6,78)_= 0.77 | ns (0.60) |
|  |  |  | Stage * Genotype * Dimension | F_(6,78)_= 1.37 | ns (0.24) |
| Repeated Measures  3-way ANOVA | Mean Incorrect Latency | D1-MSN-Blocked & WT | Genotype | F_(1,12)_= 0.24 | ns (0.63) |
|  |  |  | Dimension | F_(1,12)_= 0.31 | ns (0.59) |
|  |  |  | Genotype * Dimension | F_(1,12)_= 0.54 | ns (0.48) |
|  |  |  | Stage | F_(6,72)_= 2.83 | ***p* < 0.05** |
|  |  |  | Stage * Genotype | F_(6,72)_= 0.53 | ns (0.78) |
|  |  |  | Stage * Dimension | F_(6,72)_= 0.21 | ns (0.97) |
|  |  |  | Stage * Genotype * Dimension | F_(6,72)_= 1.90 | ns (0.09) |
| Repeated Measures  3-way ANOVA | Mean Incorrect Latency | D2-MSN-Blocked & WT | **Genotype** | **F_(1,13)_= 32.48** | ***p* < 0.001** |
|  |  |  | Dimension | F_(1,13)_= 2.53 | ns (0.14) |
|  |  |  | Genotype * Dimension | F_(1,13)_= 0.00 | ns (0.99) |
|  |  |  | **Stage** | **F_(6,78)_= 2.26** | ***p* < 0.05** |
|  |  |  | **Stage * Genotype** | **F_(6,78)_= 4.52** | ***p* < 0.001** |
|  |  |  | Stage * Dimension | F_(6,78)_= 0.91 | ns (0.49) |
|  |  |  | Stage * Genotype * Dimension | F_(6,78)_= 0.85 | ns (0.54) |
| Repeated Measures  3-way ANOVA | Omissions | D1-MSN-Blocked & WT | Genotype | F_(1,12)_= 1.14 | ns (0.31) |
|  |  |  | Dimension | F_(1,12)_= 0.00 | ns (0.99) |
|  |  |  | Genotype * Dimension | F_(1,12)_= 0.29 | ns (0.60) |
|  |  |  | Stage | F_(6,72)_= 1.54 | ns (0.18) |
|  |  |  | Stage * Genotype | F_(6,72)_= 0.87 | ns (0.52) |
|  |  |  | Stage * Dimension | F_(6,72)_= 0.56 | ns (0.76) |
|  |  |  | Stage * Genotype * Dimension | F_(6,72)_= 0.52 | ns (0.79) |
| Repeated Measures  3-way ANOVA | Omissions | D2-MSN-Blocked & WT | Genotype | F_(1,13)_= 0.00 | ns (0.96) |
|  |  |  | Dimension | F_(1,13)_= 3.70 | ns (0.07) |
|  |  |  | Genotype * Dimension | F_(1,13)_= 1.85 | ns (0.20) |
|  |  |  | Stage | F_(6,78)_= 1.87 | ns (0.10) |
|  |  |  | Stage * Genotype | F_(6,78)_= 1.05 | ns (0.40) |
|  |  |  | Stage * Dimension | F_(6,78)_= 0.29 | ns (0.94) |
|  |  |  | Stage * Genotype * Dimension | F_(6,78)_= 1.05 | ns (0.40) |
| Repeated Measures  2-Factor ANOVA | Trials To Criterion | D1-MSN-Blocked & WT | Genotype | F_(1,14)_= 0.00 | ns (0.98) |
|  |  |  | **Stage** | **F_(6,84)_= 10.78** | ***p* < 0.001** |
|  |  |  | Stage * Genotype | F_(6,84)_= 0.67 | ns (0.67) |
| Repeated Measures  2-Factor ANOVA | Trials To Criterion | D2-MSN-Blocked & WT | **Genotype** | **F_(1,15)_= 37.20** | ***p* < 0.001** |
|  |  |  | **Stage** | **F_(6,90)_= 9.22** | ***p* < 0.001** |
|  |  |  | **Stage * Genotype** | **F_(6,90)_= 5.34** | ***p* < 0.001** |
| Repeated Measures  2-Factor ANOVA | Trials To Criterion  (IDS/EDS only) | D1-MSN-Blocked & WT | Genotype | F_(1,14)_= 1.36 | ns (0.26) |
|  |  |  | **Stage** | **F_(6,84)_= 24.06** | ***p* < 0.001** |
|  |  |  | Stage * Genotype | F_(6,84)_= 1.18 | ns (0.30) |
| Repeated Measures  2-Factor ANOVA | Trials To Criterion  (IDS/EDS only) | D2-MSN-Blocked & WT | Genotype | F_(1,15)_= 2.37 | ns (0.15) |
|  |  |  | **Stage** | **F_(6,90)_= 27.85** | ***p* < 0.001** |
|  |  |  | Stage * Genotype | F_(6,90)_= 0.26 | ns (0.62) |
| Repeated Measures  2-Factor ANOVA | Mean Correct Latency | D1-MSN-Blocked & WT | Genotype | F_(1,14)_= 0.00 | ns (0.95) |
|  |  |  | **Stage** | **F_(6,84)_= 4.34** | ***p* < 0.01** |
|  |  |  | Stage * Genotype | F_(6,84)_= 0.71 | ns (0.64) |
| Repeated Measures  2-Factor ANOVA | Mean Correct Latency | D2-MSN-Blocked & WT | Genotype | F_(1,15)_= 0.17 | ns (0.69) |
|  |  |  | **Stage** | **F_(6,90)_= 3.96** | ***p* < 0.01** |
|  |  |  | Stage * Genotype | F_(6,90)_= 0.79 | ns (0.57) |
| Repeated Measures  2-Factor ANOVA | Mean Incorrect Latency | D1-MSN-Blocked & WT | Genotype | F_(1,14)_= 0.26 | ns (0.62) |
|  |  |  | **Stage** | **F_(6,84)_= 2.51** | ***p* < 0.05** |
|  |  |  | Stage * Genotype | F_(6,84)_= 0.53 | ns (0.79) |
| Repeated Measures  2-Factor ANOVA | Mean Incorrect Latency | D2-MSN-Blocked & WT | **Genotype** | **F_(1,15)_= 30.58** | ***p* < 0.001** |
|  |  |  | **Stage** | **F_(6,90)_= 2.35** | ***p* < 0.05** |
|  |  |  | **Stage * Genotype** | **F_(6,90)_= 4.67** | ***p* < 0.001** |
| Repeated Measures  2-Factor ANOVA | Omission | D1-MSN-Blocked & WT | Genotype | F_(1,14)_= 0.00 | ns (0.94) |
|  |  |  | Stage | F_(6,84)_= 2.02 | ns (0.13) |
|  |  |  | Stage * Genotype | F_(6,84)_= 1.00 | ns (0.43) |
| Repeated Measures  2-Factor ANOVA | Omission | D2-MSN-Blocked & WT | Genotype | F_(1,15)_= 1.65 | ns (0.22) |
|  |  |  | Stage | F_(6,90)_= 1.23 | ns (0.31) |
|  |  |  | Stage * Genotype | F_(6,90)_= 1.03 | ns (0.41) |
