# Supplementary figures and images for: Nucleus Accumbens Core Dopamine D2 Receptor-Expressing Neurons Control Reversal Learning but Not Set-Shifting in Behavioral Flexibility in Male Mice
Source: Front Neurosci. 2022 Jun 28;16:885380. doi: 10.3389/fnins.2022.885380 (PMC9275008; doi:10.3389/fnins.2022.885380)

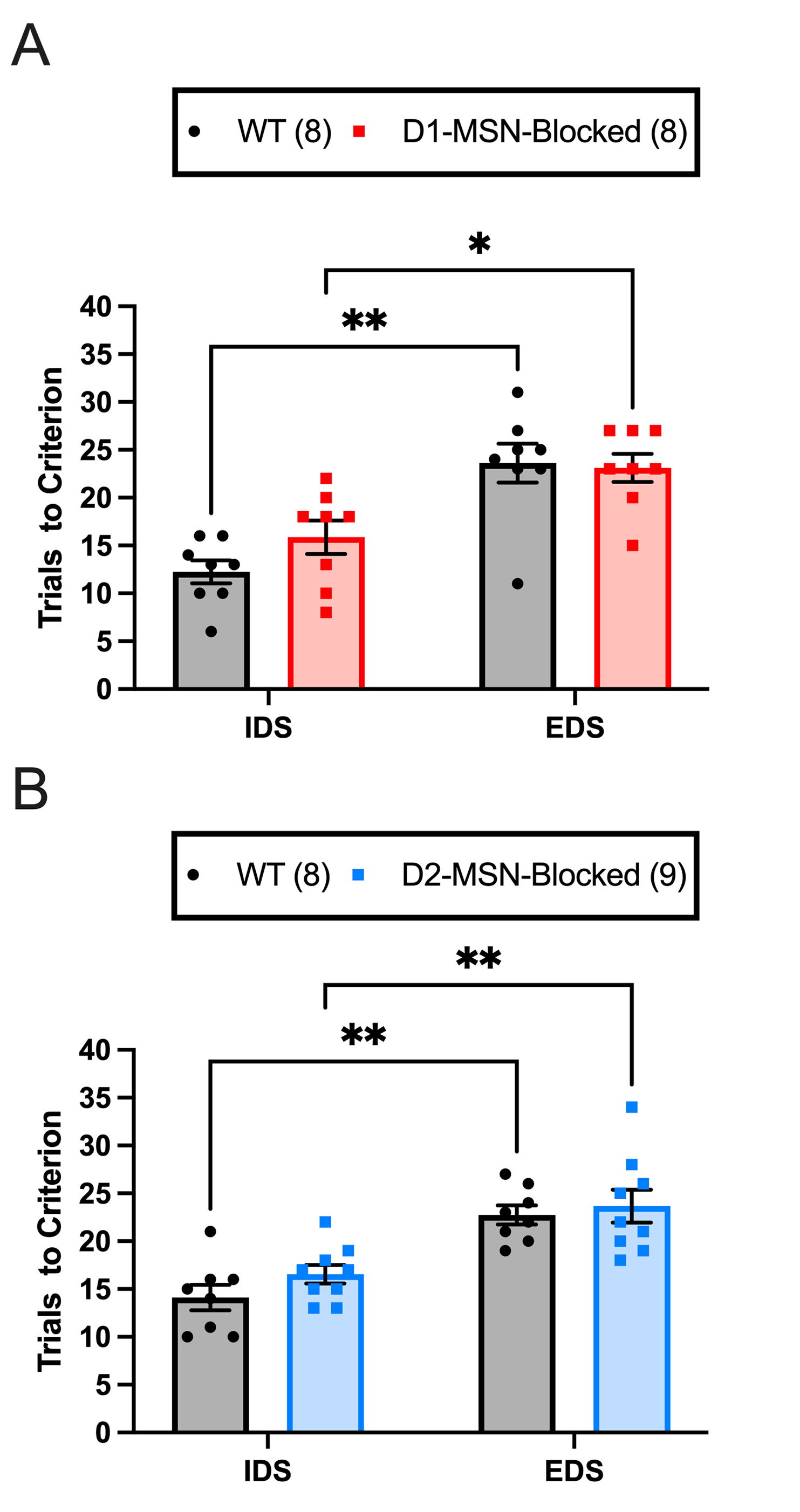

Supplement: Supplementary Figure 1 — Mice demonstrate successful formation of attentional sets. D1-MSN-Blocked (n = 8), D2-MSN-Blocked (n = 9), and WTs (n = 8, per group) took significantly more trials to reach the criterion in the extradimensional shift (EDS) stage than the intradimensional shift (IDS) stage, indicating all animals were able to successfully form an attentional set to the internal stimulus dimension that resulted in poorer performance when the set was shifted. Bars represent mean ± SEM; Bonferroni post-hoc tests (*p < 0.05, **p < 0.01). [file Image_1.TIFF]

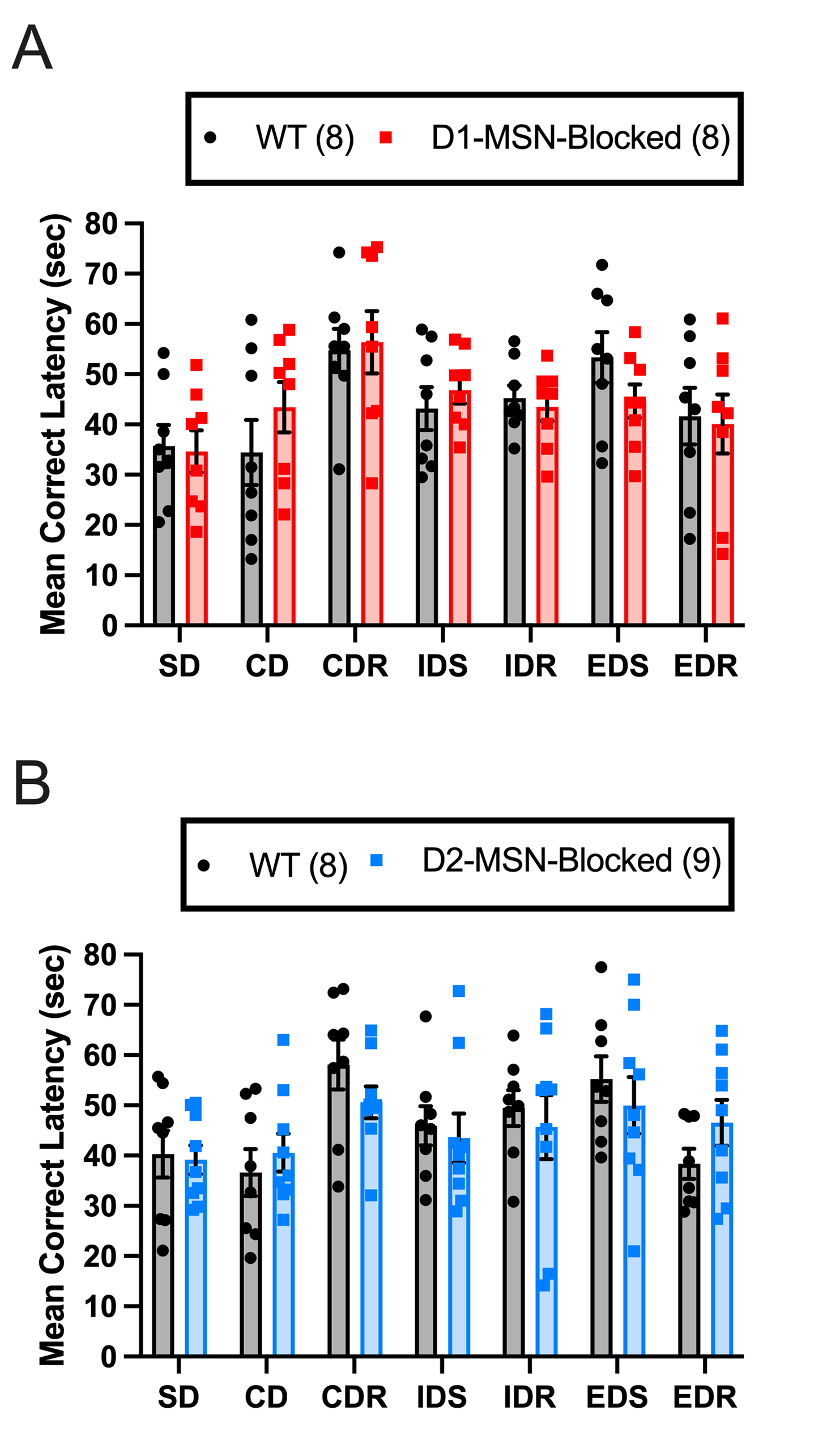

Supplement: Supplementary Figure 2 — Mean latencies to make correct responses in the attentional set-shifting task (ASST) were unaffected by neurotransmission release inhibition from NAc Core MSNs. NAc Core D1-MSN-Blocked (n = 8) (A) and D2-MSN-Blocked (n = 9) (B) mice did not significantly differ from wildtype (WT) controls (n = 8) in their mean latency to make a correct response during discrimination [simple discrimination (SD) and compound discrimination (CD)], reversal [compound discrimination reversal (CDR), intradimensional set-shift reversal (IDR), and extradimensional set-shift reversal (EDR)], and set-shifting (IDS and EDS) stages of the ASST. Bars represent mean ± SEM. [file Image_2.TIFF]

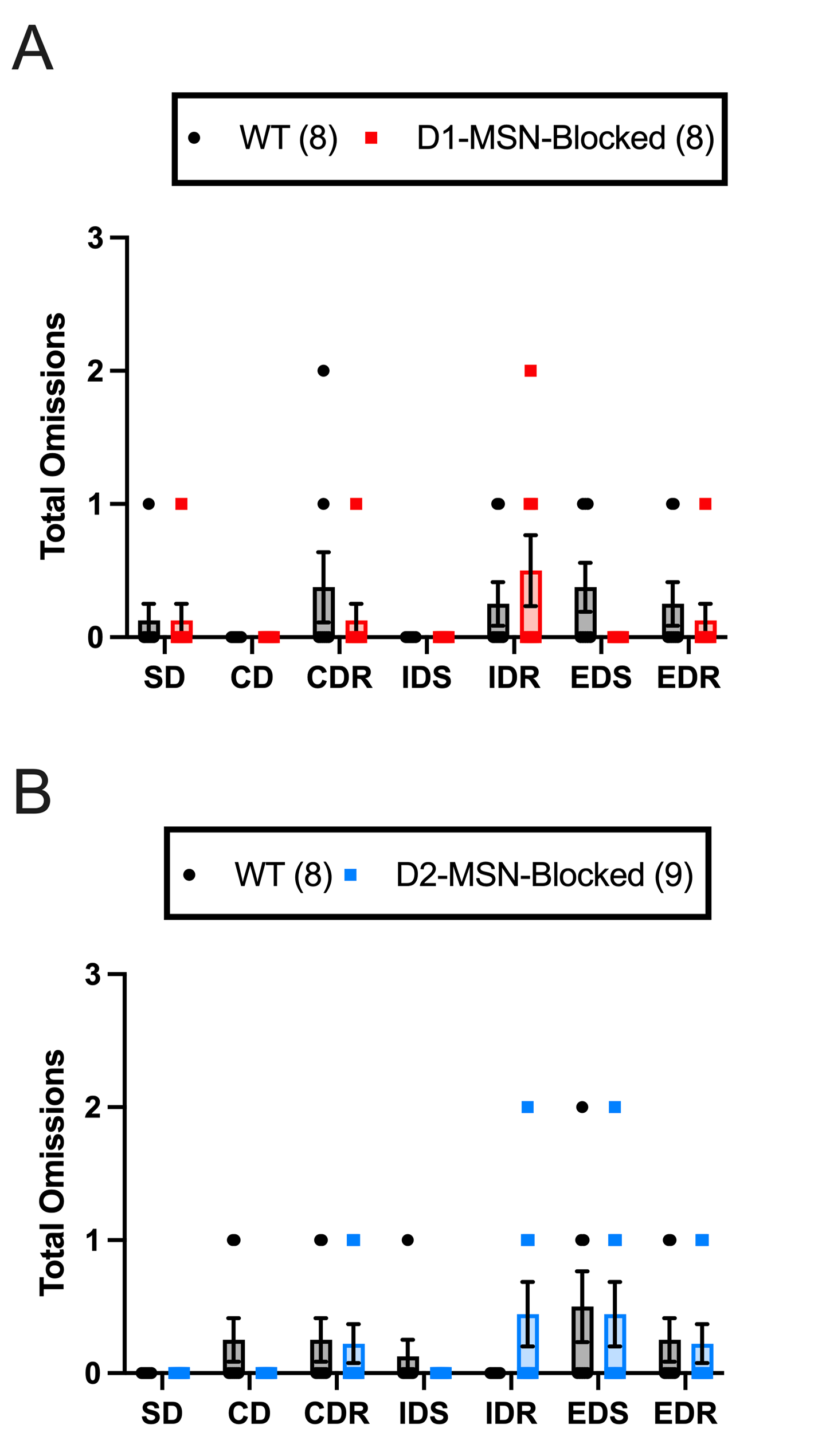

Supplement: Supplementary Figure 3 — Omission trials in the attentional set-shifting task (ASST) following NAc Core D1- and D2-MSN neurotransmitter release inhibition. The total amount of omission trials in discrimination (SD and CD), reversal (CDR, IDR, and EDR), and set-shifting (IDS and EDS) stages of the ASST did not significantly differ between WT (n = 8 per group) mice and NAc Core D1-MSN-Blocked (n = 8) (A) or D2-MSN-Blocked (n = 9) (B) mice. Bars represent mean ± SEM. [file Image_3.TIFF]

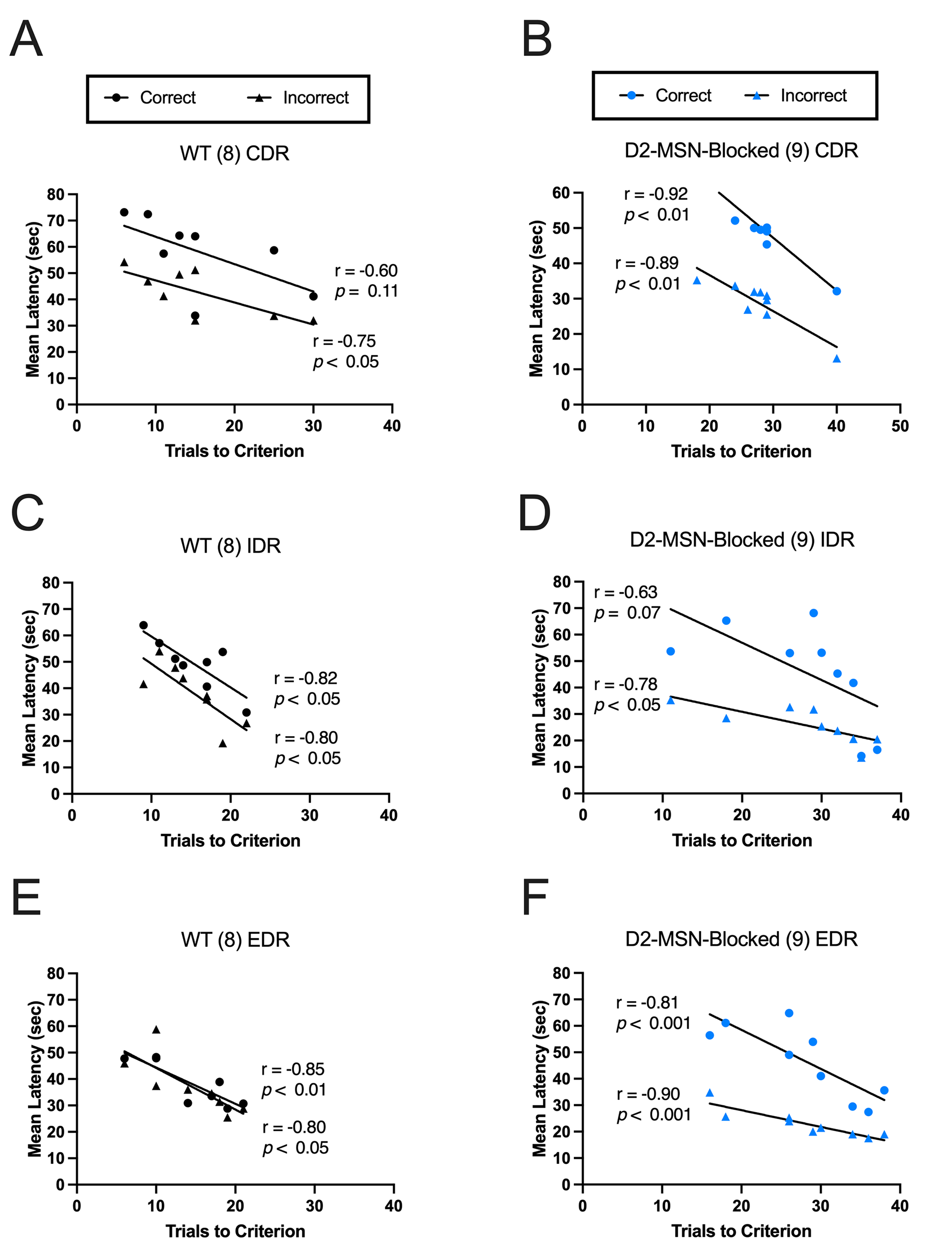

Supplement: Supplementary Figure 4 — Mean incorrect latency was negatively correlated with trials to criterion in reversal stages. In both WT (n = 8) (A,C,E) and NAc D2-MSN-Blocked (n = 9) (B,D,F) mice, the mean incorrect latency was negatively correlated with trials to criterion in all reversal stages (CDR, IDR, and EDR). Additionally, the mean correct latency was negatively correlated with trials to criterion in IDR and EDR stages in WT mice (C,E) and CDR and EDR stages in NAc Core D2-MSN-Blocked mice (B,F). Lines of best fit have been fitted to each correlation plot using simple linear regressions. Additionally, Pearson’s r values and the statistical significance of each correlation are presented. [file Image_4.TIFF]
